# M2EEG-VR: Validation of EEG Visualization and Sonification for the Detection of Neonatal Seizures on a Virtual Reality Platform

**DOI:** 10.3390/s26134167

**Published:** 2026-07-02

**Authors:** Adam Creed, Lavanya Pampana, David Murphy, Sergi Gomez, Andriy Temko, Emanuel Popovici, Andreea Factor

**Affiliations:** 1 Electrical and Electronic Engineering, University College Cork, 60 College Rd, The Lough, T12 HW58 Cork, Ireland; 118306973@umail.ucc.ie (A.C.); sgomez@umail.ucc.ie (S.G.); atemko@ucc.ie (A.T.); e.popovici@ucc.ie (E.P.); 2 MAVRIC, Computer Science and Information Technology, University College Cork, 60 College Rd, The Lough, T12 HW58 Cork, Ireland; d.murphy@ucc.ie; 3 Anatomy and Neuroscience, School of Medicine, University College Cork, 60 College Rd, The Lough, T12 HW58 Cork, Ireland; andreea.factor@ucc.ie

**Keywords:** virtual reality (VR), sonification, medical education, seizure detection, electroencephalography (EEG), VR in healthcare, biomedical signal processing, artificial intelligence (AI), machine learning (ML), human in loop AI

## Abstract

Electroencephalography (EEG) is a noninvasive tool used by healthcare professionals to measure brain electrical activity. EEG analysis can indicate various anomalies linked to different brain pathologies, including seizures. Traditionally, the analysis is confined to two-dimensional displays and relies exclusively on the visual modality, limiting a comprehensive overview. EEG analysis through visualisation is challenging and time-consuming, and artificial intelligence (AI) is increasingly used to aid the process of seizure detection. However, the educational value of AI-assisted seizure detection models depends on the explainability of the underlying models. Explainable AI can help learners understand the features and patterns associated with seizure detection and also support informed use of AI-based decision support systems. M2EEG-VR leverages the focus and immersive capabilities of virtual reality (VR) with the aim of developing a multi-modal platform for EEG seizure detection analysis with a human-in-the-loop. The ability to understand EEG and seizure patterns is key to addressing and effectively treating many neurological conditions. Neonatal seizure detection is particularly challenging where seizure patterns are subtle and context dependent. This study advances toward multi-modal analysis by encoding EEG signals into auditory representations using AI that aids in the acoustic detection of the presence of neonatal seizures in EEG. The platform also introduces a 3D brain model with a spatial mapping of seizure regions. In a user study (N = 20, 4 prior EEG experience, 16 no prior EEG experience), participants achieved higher seizure detection accuracy in the combined visual and auditory condition (mean = 7.6 ± 1.2) than in visual-only or audio-only modes. These preliminary findings suggest that a multi-modal environment may improve the accuracy of detection. However, further controlled studies are needed to ascertain the performance benefits. Usability was rated excellent (SUS = 83 ± 11), and task load remained moderate (NASA-TLX = 36.6). The findings suggest that VR multi-modal interaction can reduce cognitive load and enhance the explainability of complex EEG data in a focused virtual environment. The analysis of the diagnostic accuracy showed that participants without prior EEG knowledge performed similarly across all modalities to those with prior EEG knowledge. This implies that the accessibility barrier is reduced for novice users using the tool for the EEG review/detection task. This, together with high usability and moderate task load scores, indicates that the tool may be suitable for medical training applications. A multi-modal EEG in VR may prove useful in education and also be used as a test bench to further explore AI with human-in-the-loop paradigms for seizure detection.

## 1. Introduction

Electroencephalography (EEG) is an important tool in medical education and neurophysiology research that allows noninvasive measurement of electrical activity in the brain. EEG plays an essential role in the diagnosis and monitoring of neurological conditions, including epilepsy [1], sleep disorders [2], and Alzheimer’s disease [3]. Meticulous analysis of EEG is especially critical in neonatal EEG analysis, where immediate and accurate detection of seizures can have a profound impact on treatment and outcomes [4].

Seizures are sudden, uncontrolled electrical disturbances in the brain that can affect behaviour, movement, or consciousness [5,6]. They are a manifestation of various neurological conditions, including epilepsy [7], traumatic brain injury [8], and stroke [9]. In neonatal care, seizure detection is more difficult due to the subtle and atypical presentation of seizures in the population. Neonatal seizures can have long-term consequences on cognitive and motor development, making early and accurate detection imperative for timely intervention [10].

Traditional EEG analysis has been the gold standard for neonatal seizure detection, as it provides detailed information on the electrical activity of the brain [11]. The physiological characteristics of the neonatal brain, as well as the lack of clinical signs to accompany seizures, make the EEG analysis particularly challenging. This complexity is heightened by the need to interpret multiple data channels that represent activity in various regions of the brain [12,13]. Detection of seizures through visualisation is time-consuming and requires scrolling through hours of multi-channel recordings on a 2D screen [14]. This underscores the need for tools that enable more efficient and accurate analysis through improved focus during the analysis period or the provision of efficient attention mechanisms.

Historically, most EEG research has focused on improving data analysis and interpretation using advanced signal processing techniques [15], machine learning (ML), and artificial intelligence (AI) [16]. These approaches have led to significant progress in areas such as seizure detection [17]. Traditional 2D displays remain the standard, despite their inherent limitations in representing the complex spatial and temporal dynamics of brain activity. As a result, the potential to utilise focused and immersive three-dimensional (3D) visualisation remains largely underexplored. This study explores whether a multi-modal VR-based seizure analysis is feasible and usable for medical education.

Advances in the visualisation of EEG have inspired the development of interactive tools for neurophysiological data. For example, EEGNET, an open-source software package developed for Matlab that supports magnetoencephalography (MEG) and EEG recordings, analyses functional connectivity and provides visualisation of the brain network [18]. Similarly, Balloni et al. introduced the brain visualizer that integrates both EEG and magnetic resonance imaging (MRI) data to reconstruct a 3D brain activity visualizer to observe spatial activation patterns [19]. This study is the closest to exploring the development of an EEG and MRI-based brain visualisation tool in a VR platform. Although the process of pre-processing EEG data, more specifically, converting EEG data to a csv file, is similar, the study does not explore seizure detection and audio encoded EEG. In 2018, Steffert explored the potential of real-time EEG sonification, suggesting that auditory representations can improve temporal sensitivity in EEG-based tasks [20].

Although Turan [21] and Wu [22] combined epilepsy research and VR, these studies focused mainly on educating and training parents and nurses in the management of seizures. These works underscore the importance of spatial and multi-modal representations in EEG seizure analysis but remain limited to visualisation, connectivity mapping, or seizure management. In contrast, the present study integrates real-time EEG analysis, 3D visualisation and auditory sonification within an immersive virtual reality environment specifically designed to provide a platform that can be useful for understanding complex EEG-based seizure analysis and help medical students engage effectively with AI-assisted seizure analysis tools [23].

By focusing on interactive and immersive visualisation in VR, a critical gap in EEG research can be addressed, providing medical students and healthcare professionals with tools that potentially enhance understanding of complex brain waveforms and support human-in-the-loop AI decision-making. In recent years, advances in VR technology have opened up new possibilities for data visualisation, providing a platform to represent multidimensional data in a spatially immersive environment [24]. VR offers unique opportunities to transform the interpretation of EEG by allowing users to interact spatially with the data, thus reducing cognitive load and improving pattern recognition [25].

Recent advancements in signal processing, machine learning, and AI have significantly improved automated seizure detection capabilities [26,27,28]. However, AI-only models often lack explainability, creating barriers to adoption in medical education and training [29]. A method of analysis through EEG sonification using AI as an attention mechanism to speed up EEG analysis is proposed by [16]. This allows users to hear the electrical activity of the brain as a dynamic auditory experience. When used as a complement to visualisation, this sonification technique not only provides an additional sensory modality for interpreting EEG data, but also improves the users’ ability to detect patterns and anomalies in brain electrical signals that may be visually overlooked as one-dimensional time series data. Moreover in [30], parametric EEG sonification has proven useful in Alzheimer’s detection suggesting its potential for broader complementary and accessible method for examining brain patterns in neurodegenerative diseases. Hence, it was integrated into this platform. Furthermore, the results of the AI model [31] provide seizure zones in the form of feature maps. These feature maps, detected from seizure events are projected onto a 3D brain model, offering a spatial representation of potential seizure zones. The ability to visualise these detections within a 3D brain framework may help interpret seizures better and may facilitate familiarity with AI-assisted decision-making. Implementations of AI-assisted neonatal EEG sonification, including those in [32,33], show the feasibility of real-time or low-power deployments that complement the proposed framework. Such innovations are particularly promising in neonatal care, where time-sensitive and accurate detection of seizures can substantially improve outcomes [34].

The primary objective of the study is to investigate the challenges and opportunities of a multi-modal EEG analysis framework in medical education that support learning, interpretation, and increase familiarity with AI assisted tools. This goal has led to the design and development of a VR platform for neonatal EEG seizure analysis that utilises the traditional analysis through visualisation, explainable AI through AI-assisted sonification, signal processing, and 3D visualisation of the brain, contributing to a multi-modal analysis of seizure detection. By integrating all these features under the same platform, the user is exposed to an immersive environment in which attention is enhanced by the use of VR and AI. Steps were taken to ensure that the virtual environment presents a natural, immersive, and intuitive framework for the end users.

Design, development and usability of a neonatal EEG analysis platform within a VR environment is discussed in this paper. The platform re-imagines how EEG data can be presented, leveraging the immersive capabilities of VR to offer a fully 3D environment where users can interact with and explore brain activity data in a focused, immersive, and multi-sensory approach with a human-in-the-loop approach. To validate the effectiveness of this VR-based EEG platform, a series of user evaluations was conducted. Participants, including users with and without prior EEG experience, interacted with the system and provided feedback on its usability and functionality.

The following are the main contributions of the paper:

- Development of a 3D virtual environment that allows visualization of EEG time series data in a focused immersive context.
- Integration of sonification techniques from [16] that use an AI algorithm from [31] to represent EEG signals audibly, greatly reducing the time to detect seizures.
- Incorporation of visualization of detected seizures with a 3D brain model.
- Design with high user-experience (UX) and usability leading to a user-friendly interactive VR environment with intuitive navigation.

## 2. Materials and Methods

### 2.1. Virtual Environment and System Setup

The development of the VR-based EEG visualisation platform was undertaken with a carefully selected suite of hardware and software tools optimised for real-time immersive visualisation, high-performance computation, and user-friendly interaction. Each component was chosen to ensure the platform’s responsiveness, compatibility, and ability to deliver an intuitive user experience.

The primary hardware used in this study was the Pico Neo 3 Pro VR headset [35]. This device was selected due to its high-resolution display (3664 × 1920 resolution, 773 PPI) and 90Hz refresh rate, which contribute to a clear and smooth VR experience. The headset’s standalone processing capability, powered by a Qualcomm Snapdragon XR2 processor, provided considerable computational power while maintaining a lightweight, wireless design. This portability enables clinicians and researchers to interact with the platform untethered, improving usability in clinical settings.

The development environment for the VR application was Unity 3D (version 2023.2.4f1) [36], a widely used game engine known for its robust support of VR applications and compatibility with a wide range of VR hardware through the OpenXR standard [37]. Unity’s powerful 3D rendering capabilities facilitated the detailed visualisation of EEG data on a 3D brain model, while its support for *C#* [38] scripting allowed for the development of interactions and real-time processing features specific to EEG data visualisation.

### 2.2. Implementation

#### 2.2.1. Data Visualisation and Interaction Modules

The VR-based EEG visualisation platform relies on a combination of two modules to provide an interactive, immersive, and multi-modal experience for users. Two key components in this setup are the EEG visualization module, developed specifically for this platform, and the integration of the sonification algorithm from Gomez-Quintana et al. [16] and its edge implementation by O’Sullivan et al. [32]. The EEG data displayed was taken from the publicly available open source Helsinki dataset [39]. The selection of 10 EEG epochs was random from the set of epochs in which all three annotators agree on the outcome (at least one seizure present or no seizure).

Data pre-processing was conducted in Python [40], leveraging libraries such as NumPy [41], and Pandas [42]. Python’s extensive scientific computing ecosystem provided the necessary tools for handling and transforming EEG data into a comma separated values (CSV) file. The Python-based pre-processing pipeline included signal filtering, artifact removal, and probability output ensuring that EEG data were formatted and optimised for rendering in VR. The pipeline generates a single CSV file containing the original EEG data, the frequency bands of each EEG channel and the probability of seizure for each brain region. A summary of the format of the CSV file is shown in Table 1.

To enable smooth EEG visualisation in VR, the CSV data is pre-loaded onto the headset, eliminating the need for continuous data transfer, reducing the computational and system load and ensuring stable performance without dropping any rendered frames. By offloading data processing to external systems, the VR headset can focus solely on rendering and reducing latency. This approach maintains a fluid user experience, even with complex EEG datasets, making analysis and visualisation more effective in VR.

To effectively represent EEG data within a three-dimensional environment, a dedicated EEG visualisation module was developed. This module is designed to process and render EEG signal amplitudes onto a 3D plane, giving an intuitive and immersive view of neural activity. Initially, each EEG channel is displayed as a 2D waveform, mirroring traditional EEG visualisations for ease of reference. The data is then mapped onto a 3D plane where each channel is decomposed into its frequency bands. This multi-dimensional approach enables users to examine EEG data from varied perspectives, deepening their insight into neural dynamics while using the familiar 2D waveform format for display. The Main Screen of the VR platform was designed to closely resemble the conventional 2D EEG analysis interface. The primary goal of this screen was to offer users a familiar environment, encouraging them to engage with the software while also introducing innovative ways to interact with the data. By maintaining a familiar layout, users experienced minimal disruption when transitioning from conventional tools to this VR-based platform.

#### 2.2.2. Sonification of EEG Data

To improve the interpretability of EEG data, a sonification algorithm [16] was integrated in conjunction with the visual representation. This feature converts EEG timeseries recordings into auditory cues, allowing users to “hear” neural activity patterns. The sonification method maps variations in EEG signals to specific audible sound frequencies (including frequencies in the scream domain where human hearing is most selective), enabling users to identify patterns such as rhythmic fluctuations or slow evolution in time of frequencies associated with seizure activity. The sonification process is incorporated into the data pre-processing stage, where an algorithm generates a .wav file corresponding to the EEG data. This audio file is then preloaded into the VR headset along with the visualised EEG data, creating a cohesive multi-modal experience that facilitates EEG analysis within an immersive VR environment. Human hearing is especially equipped to distinguish the slow evolution of the frequencies associated with seizures over time. In our system AI acts as an attention mechanism for human hearing, combined they provide a performance greater than that of AI on its own [16].

**Figure 1:**
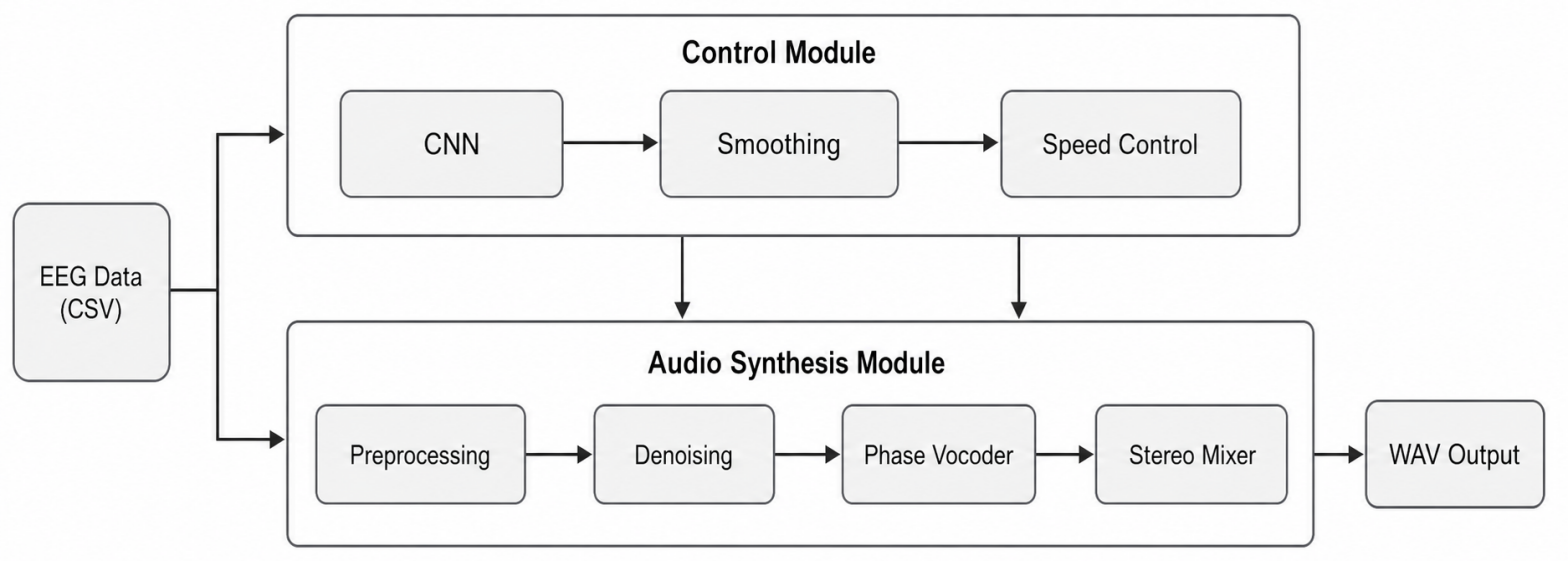
Flowchart of the main blocks of the EEG AI-assisted sonification algorithm.

The sonification algorithm is described in Figure 1. It accepts 8-channel neonatal EEG recordings as input and generates stereo audio as output. It is composed of two modules: the Audio Synthesis Module and the AI Control Module. Within the Audio Synthesis Module, EEG signals from the eight channels undergo preprocessing and denoising (to reduce noise and remove artifacts, particularly electrocardiogram (ECG) interference) before a phase vocoder converts their frequency content into the audible range with a dynamic time-stretching factor informed by a deep learning model to highlight seizure events. The resulting audio signals are then merged by a stereo mixer into two output channels, reflecting the relative positions of the electrodes (left-right cerebral hemispheres positioning to left–right stereo). The AI Control Module analyzes successive EEG segments and outputs, at one-second intervals, the probability of seizure activity for each segment. These probabilities govern a variable time-compression rate that depends on seizure likelihood, thereby acting as an AI-driven attention mechanism that shapes the perceived audio. The higher the likelihood of seizure, the longer the audio generated allowing improved focus on the corresponding segments of EEG. This feature allows for both speed of analysis as well as improved attention for the listener. Remarkably, human-interpreted AI-assisted sonification is found to be more accurate than AI analysis alone [16] due to human ability to perceive slow evolution in time of sound corresponding with the slow evolution in time of sezures. The proposed VR framework allows for both fixed sonification as well as variable sonification. For the purpose of this study we considered only the AI assisted sonification in which the speed of sonification is variable, depending on the probabilities of the CNN.

#### 2.2.3. 3D Brain Model and Spatial Seizure Visualisation

A core feature of the VR-based EEG visualisation platform is the 3D brain model, which dynamically changes colours based on probability data from the generated CSV file, indicating regions with heightened neural activity and seizure likelihood. This colour-mapping approach provides an intuitive visual cue, enabling clinicians and researchers to quickly assess areas of concern within the brain.

The EEG pre-processing pipeline generates probability values for each brain region, which are stored in a CSV file. These probabilities, representing the likelihood of seizure activity or other neural events, are mapped to specific brain regions within the 3D model. Each EEG channel’s data is aligned with a corresponding brain region, ensuring that the probabilities are accurately represented spatially.

The colour-coding scheme is designed for clarity in data interpretation:

- Green denotes lower probabilities (<10%), indicating baseline or typical neural activity.
- Yellow indicates moderate probabilities (10–50%), signaling regions that may warrant further observation.
- Red highlights high-probability areas (>50%), marking them as regions of elevated concern.

Colour selection is guided by conventional brain mapping software that uses red, yellow, green, and blue to signify a gradient of activity, helping clinicians immediately identify abnormal brain activity. This real-time colour mapping provides a visually intuitive and rapid overview of neural activity probabilities, allowing clinicians and researchers to efficiently identify and prioritise high-risk regions for further analysis, thus improving the interpretability and diagnostic potential of EEG data in VR.

### 2.3. Testing and Participant Preparation

To ensure the development of a robust VR platform, internal testing was performed within the research group focused on identifying and addressing significant technical issues at an early stage of development. Members engaged with the platform and provided essential feedback regarding its functionality, user interface, and overall usability. This feedback was crucial for implementing early refinements, such as enhancements to the user interface for improved navigation and integration of EEG data within the VR environment.

The study involved a total of 20 University College Cork (UCC) participants, including 4 with prior EEG experience and 16 participants with no prior experience in EEG interpretation. At the beginning of the study, participants were given a brief introduction to VR, the objective of the study, EEG and seizure characteristics (for visual and audio inspection), and an overview of the steps of execution. Participants were asked to complete a series of tasks that included EEG analysis, seizure detection, and platform interaction, followed by a post-experiment questionnaire to assess their experiences and satisfaction with the platform. Participants were asked to assess if a seizure was present or not in randomly selected 10 EEG epochs from the Helsinki neonatal EEG Database. Five of the epochs contained at least one seizure. All epochs were annotated by 3 experts, and for the 10 selected epochs, all annotators agreed on their labels. The selected 10 epochs were first visually analysed, then the 10 sonified representations (audio) were listened to, and finally, for each epoch, the participants could navigate back and forth between both visualisation and sonification. The same epoch ordering is maintained for each of the analyses. For each modality and for each epoch, an answer “Yes” was recorded if a seizure was present, or “No" was recorded if there was no seizure present. The maximum score for each participant is 10, corresponding to correct classification of the 10 epochs.

The iPQ (iGroup Presence Questionnaire) [43,44] questionnaire was provided to all participants to assess how immersion VR impacts EEG data interpretation in a 3D virtual environment. In the context of medical and scientific visualisation, the iPQ has been employed to evaluate the effectiveness of VR-based training and assessment tools. The NASA Task Load Index (NASA-TLX) [45] was employed to assess the cognitive workload imposed on users while interacting with the VR-based EEG visualisation platform. Participants were also requested to complete a System Usability Scale (SUS) [46] to gather comprehensive feedback on the platform’s usability, focusing on elements such as navigation ease, interface intuitiveness, and overall user satisfaction. A general questionnaire was also provided to gather broader feedback on the platform’s usability, including aspects such as ease of navigation, intuitiveness of the interface, and overall user satisfaction. Users were also asked to provide qualitative feedback, highlighting any difficulties encountered and suggestions for potential improvements.

**Figure 2:**
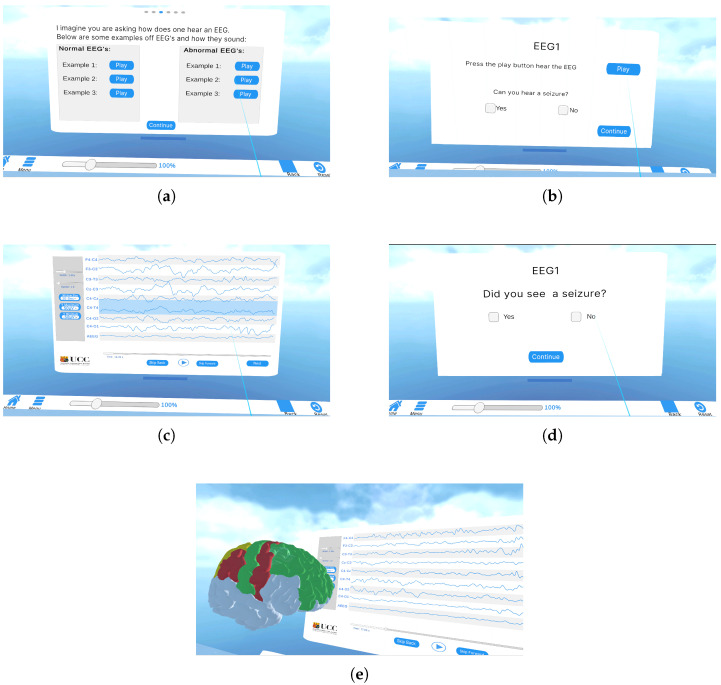
Representative panels from the VR-based EEG experiment. Panels show examples of audio and visual EEG samples, AI-assisted EEG sonification, EEG waveform visualisation, visual seizure examination, and 3D brain-based seizure intensity visualisation. (**a**) Examples of audio and visual EEG samples. (**b**) Examination of audio EEG using AI-assisted sonification. (**c**) Eight-channel EEG montage showing representative waveforms. (**d**) Visual examination of EEG waveforms. (**e**) 3D brain visualisation of seizure intensity.

Figure 2 presents representative stages of the VR-based EEG experiment, including audio, visual, and combined EEG interaction views.

## 3. Results

The effectiveness and performance of the VR-based EEG visualisation platform were evaluated through two main studies: User Experience Testing and Seizure Detection Testing. This section presents results and findings from each study, emphasising the platform’s usability, detection accuracy, and system performance across different conditions and hardware configurations. These steps allow for an easy-to-follow, versatile flow of EEG analysis, combining traditional visualisation with sonification and 3D brain visualisation of seizure activity.

### 3.1. User Experience Testing

The user experience testing involved assessing the platform’s usability, ease of interaction, immersion, and cognitive load.

Descriptive Statistics: Table 2 presents key descriptive statistics for the usability and experience measures, providing insights into participants’ interactions with the VR-based EEG visualisation platform.

The NASA-TLX [45] workload ratings highlighted important aspects of participant experience. Mental workload yielded a mean score of 8.90 (σ=5.20), reflecting moderate cognitive demand, with scores ranging from 1.5 to 15.5, indicating variability in participants’ perceived difficulty. Physical workload was lower, with a mean of 4.85 (σ=3.77), suggesting minimal physical exertion, aligning well with the requirements of a VR-based task. Temporal workload had a mean of 3.95 (σ=3.36), implying that participants did not experience significant time pressure, with scores spanning 1.0 to 12.5. Performance ratings, with a mean score of 6.20 (σ=3.93), indicated that participants generally perceived themselves as effective in task completion, with scores ranging from 2.0 to 16.5. Effort ratings averaged 7.83 (σ=3.69), reflecting moderate levels of exertion required to interact with the platform. Frustration levels averaged 3.92 (σ=3.56), with scores ranging from 0.0 to 12.0, suggesting that while the platform was cognitively engaging, it did not elicit high levels of frustration for most participants.

The System Usability Scale (SUS) [46] results demonstrated strong user satisfaction, with a mean score of 83.00 (σ=11.14). This score, categorised as “excellent” [47], underscores the platform’s high usability. Scores ranged from 60.0 to 100.0, reflecting consistently positive user experiences.

The iPQ (Igroup Presence Questionnaire) [48] provided an assessment of presence within the VR environment. The Spatial Presence subscale recorded a mean score of 13.25 (σ=1.62), indicating that participants experienced a robust sense of immersion, which is critical for effective EEG data interpretation. The Involvement subscale had a mean of 17.40 (σ=2.84), demonstrating high levels of engagement during tasks. Realism, measuring the perceived authenticity of the VR environment, yielded a mean score of 16.40 (σ=4.36), indicating that participants generally found the environment to be credible and realistic. The General Presence dimension had a lower mean score of 1.90 (σ=1.02), reflecting variability in the overall sense of immersion across participants.

**Figure 3:**
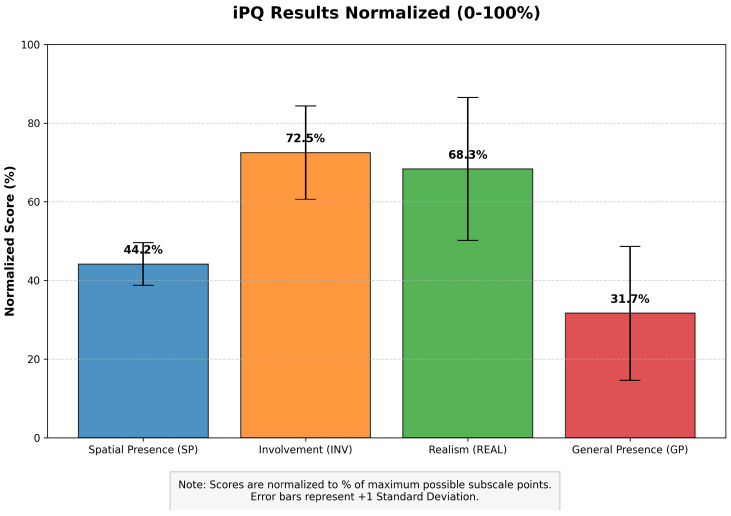
iPQ results normalized scores.

These findings, as can be seen in Figure 3 illustrate that the VR platform successfully balances usability, cognitive engagement, and immersion. The results suggest that the platform holds significant potential as an innovative and user-friendly tool for EEG data visualisation and interaction in VR environments.

Participants provided qualitative feedback through open-ended post-experiment questions, offering insights into their experiences with the VR-based EEG visualisation platform. The feedback highlighted several key themes, including strengths and areas for improvement. Participants found the VR interface intuitive, with the 3D manipulation of EEG data aiding in understanding complex patterns. Participants appreciated features such as the ability to customise screen layouts for better visualisation, though some noted that more guidance on these features was needed, underscoring the importance of an enhanced walkthrough for new users. The immersive VR environment was praised for improving focus and engagement. However, some participants found the brightness uncomfortable and noted the inability to see their surroundings, suggesting the potential for moving towards augmented reality (AR) or mixed reality (MR) for improved usability and comfort. A recurring issue was the inconsistent functionality of interface buttons, particularly dropdown menus and sliders, due to small clickable areas. Participants suggested enlarging these elements to facilitate easier interaction and reduce complexity. This feedback highlights the platform’s strengths in usability and immersion, while also pointing to specific areas for refinement. Addressing these issues will enhance the platform’s effectiveness and accessibility, ensuring a more engaging and user-friendly experience for EEG data visualisation.

### 3.2. Seizure Detection Testing

The clinical diagnosis testing focused on evaluating the platform’s ability to support accurate interpretation of EEG data, specifically in detecting seizure events. Participants analysed EEG recordings across different modalities, namely sound only, visualisation only, and combined sound and visualisation, and provided interpretation for each group in detecting seizure events. The performance was measured by comparing their accuracy against the expert-labelled ground truth from the Helsinki dataset.

Table 3 presents descriptive statistics for diagnostic performance across the sound, visualisation, and combined modalities. The highest diagnostic accuracy was observed in the Combined modality (mean = 7.60, σ=1.23), followed by visualisation (mean = 6.10, σ=1.25), and Sound (mean = 5.50, σ=1.28). This result supports the hypothesis that combining auditory and visual signals enhances diagnostic performance by providing complementary information about EEG activity. A repeated measures ANOVA indicated a significant difference in performance scores across the three modalities (*p* < 0.001). Post-hoc paired t-tests confirmed that the combined modality (mean = 7.60) significantly outperformed both sonification (mean = 5.50; *p* < 0.001) and visualisation (mean = 6.10; *p* < 0.001) modalities. Furthermore, the magnitude of these improvements was substantial, yielding large effect sizes for both the combined modality vs. sonification comparison (Cohen’s d = 1.68) and the combined modality vs. visualisation comparison (Cohen’s d = 1.21) as presented in Table 4. Table 5 presents the results of the one-way repeated-measures ANOVA examining the effect of modality on detection accuracy. The analysis revealed a significant main effect of modality, F(2,38)=22.30, p<0.001, with a large effect size (partial $\eta^{2}=0.54$). The between-methods mean square (MS=23.40) was substantially larger than the residual mean square (MS=1.05), exceeding the critical value of F=3.24 at α=0.05. These findings indicate that detection accuracy differed significantly across the three modality conditions, supporting the use of pairwise comparisons reported in Table 4. IBM’s SPSS software was used for the statistical analysis.

Comparative analysis between participants with and without prior EEG knowledge. An analysis of the diagnostic accuracy showed that participants with prior EEG knowledge performed similarly across all modalities to those without it. The group with prior EEG knowledge achieved a slightly higher mean score in the combined modality (mean = 8.00, σ = 1.15) compared with the group without prior EEG knowledge (mean = 7.50, σ = 1.26). However, this difference was not statistically significant (p=0.48).

## 4. Discussion

This study investigates whether immersive audio–visual encoding of EEG in VR improved human interpretation of EEG in the context of seizure detection for neonates. The EEG signals can be both seen and heard, and the goal of the study is to determine if study participants notice patterns more quickly and accurately in the focused and highly immersive VR platform. Only a subset of 10 of the available EEG epochs (a total of 79) were considered in this initial implementation. Also, only epochs in which all three original annotators agree on the outcome were considered to simplify our evaluation also given the fact that we had participants with previous EEG experience while most participants had no prior EEG experience.

The results in all testing categories confirm the effectiveness and versatility of the VR-based EEG visualisation platform. The high SUS score and the low cognitive workload ratings demonstrate the usability of the platform, while seizure detection testing results indicate that multi-modal EEG visualisation significantly improves diagnostic accuracy for the participants in the study.

Traditionally, EEG is observed as 2D data on a flat screen, and trained healthcare professionals have to scroll long pages of data to monitor and detect seizures. Simplifying this process with AI helps negate human-induced errors and also provides faster results as opposed to long hours of manual detection. This study utilises the state-of-the-art AI seizure detection model developed for newborns that has an AUC of 98.5% [31]. Though the results of AI-based detection methods are remarkable, it still lacks explainability. In a recent survey study [49], 74% of medical respondents attributed the lack of training and the black box nature of automated seizure detection tools as one of the main barriers to using existing automated EEG seizure detection tools. Explainability in AI (XAI) is an active area of research, and researchers suggest a combination of human-in-the-loop (HITL) and XAI methods to solve the problems that might arise due to the integration of AI in real-world applications [50]. The latency in seizure detection was also mentioned as one of the main reasons, with an accepted latency of less than 15 s from most of the respondents as a barrier to the adoption of AI-based automated seizure detection tools [49].

This study uses audio-coded EEG, which changes how we understand EEG. EEG signals are first automatically segmented into seizure or non-seizure intervals using a deep learning model. The resulting segments are subsequently mapped to audio using sonification, where in the seizure segments are selectively time stretched to facilitate auditory inspection [16]. The VR platform designed in this study adopts a human-in-the-loop paradigm. Furthermore, it leverages the innate ability of humans to differentiate between high-pitched sounds, also known as frequency selectivity, “a key characteristic of the human auditory system and is particularly sharp when compared to other mammals” [51]. Another important feature is the ability of hearing (slow) frequency evolution in time, which is a characteristic of seizures that is sometimes difficult to capture even by AI. Finally, a key motivation for using audio encoded EEG is that it compresses long hours of recordings into seconds of audio, enabling rapid review of long EEG data.

Integrating all information within the same platform allows for further investigation into what type of EEG epochs lead to issues for the participants when it comes to visualisation and sonification modalities. Seizures can be very short and in this case sonification might struggle, or artifacts can sometimes affect the analysis through visualisation or indeed AI might incorrectly attribute high probabilities of seizure to epochs which have no seizure. These modalities combined provide a valuable medical/physiology education tool. This multi-modal approach can be a first step towards analysing very complex EEG epochs (for which even experienced annotators might disagree).

There are some limitations to this study. First, it was conducted with a small sample size of participants, which may limit the generalisability of the results. Only a subset of epochs was considered in this study, those in which all annotators agree. Future research should include a larger and more diverse sample of participants and data to validate the effectiveness of the platform in different settings and user groups. Another limitation is the experimental design may not have fully controlled for the learning or familiarization effects and so, randomized or counterbalanced design will be adopted in the future studies. A special focus could be derived for particularly difficult to detect seizures, for example those in which only 2 out of 3 annotators agree. Furthermore, the study did not address the long-term usability and sustainability of the platform, which will be critical for its adoption and integration into clinical education and research workflows. Future work should focus on addressing these limitations to ensure that the platform meets the needs and requirements of its users. In general, the feasibility study demonstrated that the VR-based EEG visualisation platform offers a potential solution to improve the interpretation of EEG data in educational and research settings. The results of the feasibility study suggest that the platform may be suitable to support EEG data analysis, providing an intuitive, engaging, and usable tool to visualise brain activity in a spatially rich 3D environment. By addressing key factors such as usability and accuracy, the platform has the potential to transform the interpretation of EEG data and support a wide range of applications in education, medical research and healthcare. The feasibility study has laid a foundation for future work on the platform, highlighting its scalability, accessibility, and potential impact on EEG analysis, human in loop decision-making and primarily aiding in medical education.

## 5. Conclusions

This study presents a VR-based platform for immersive EEG visualisation, designed to enhance the interpretability of EEG data, particularly for critical EEG review tasks such as neonatal seizure detection. By integrating 3D visualisations, sonification, and AI-driven seizure detection within an intuitive VR environment, the platform offers a multi-modal approach to EEG analysis with an emphasis on usability.

The results from user experience testing demonstrate that the platform is both accessible and highly usable, with a strong sense of engagement and minimal cognitive load reported by participants. The high System Usability Scale (SUS) score and moderate NASA Task Load Index (NASA-TLX) ratings indicate that the VR environment provides a user-friendly and engaging experience, supporting both users with and without prior EEG knowledge in analysing complex EEG patterns. Furthermore, the data suggest that combining visual and auditory modalities has the potential to enhance detection accuracy, with the multi-modal presentation yielding significantly higher performance compared to single-modality conditions. This suggests that the platform could be particularly beneficial in medical education and training.

This VR-based EEG visualisation platform contributes to the neuroscience research, leveraging VR’s immersive capabilities to create a spatially rich and intuitive environment for EEG interpretation. By providing users with both visual and auditory insights and supporting human in the loop seizure detection, the platform has the potential to improve accuracy and user engagement further. Future work may explore enhancements to the AI-driven seizure detection model, as well as further integration with neuroimaging modalities to provide a comprehensive analysis tool. Additionally, further testing with larger sample sizes will provide valuable feedback to refine the platform’s functionality and broaden its applicability.

In conclusion, this VR-based EEG platform introduces an innovative, immersive approach to EEG analysis that may significantly benefit both medical students and researchers. Through further development and validation, it may support more efficient and accurate interpretations of complex neural data.

## Figures and Tables

**Table 1:** Organisation of EEG-derived features and seizure probability outputs in the CSV file. The rows are grouped according to raw EEG channels, AI-generated seizure probability outputs, amplitude EEG, and frequency-band-specific EEG features.

Row Number (s)	Data Type
1–8	Normal EEG data
9–16	Seizure probability outputs
17	Amplitude EEG
18–25	Delta-band features
26–33	Theta-band features
34–41	Alpha-band features
42–49	Beta-band features
50–57	Gamma-band features

Note: EEG = electroencephalography. Row ranges indicate the corresponding organisation of data streams within the CSV file.

**Table 2:** Descriptive statistics for NASA-TLX, SUS, and iPQ measures. Values are reported as mean and standard deviation.

Measure	Mean	Standard Deviation
**NASA-TLX**		
Mental workload	8.90	5.20
Physical workload	4.85	3.77
Temporal workload	3.95	3.36
Performance	6.20	3.93
Effort	7.83	3.69
Frustration	3.92	3.56
**System Usability Scale (SUS)**	83.00	11.14
**iPQ**		
Spatial presence	13.25	1.62
Involvement	17.40	2.84
Realism	16.40	4.36
General presence	1.90	1.02

Note: NASA-TLX = National Aeronautics and Space Administration Task Load Index; SUS = System Usability Scale; iPQ = Igroup Presence Questionnaire.

**Table 3:** Descriptive statistics for detection accuracy across the three modalities audio only, visual only and audio + visual (n=20). Values are reported as mean, standard deviation (SD), median, and 95% confidence interval (CI).

Condition	N	Mean	SD	Median	95% CI
M1 (Audio only)	20	5.50	1.28	6.0	[4.94, 6.06]
M2 (Visual only)	20	6.10	1.25	6.0	[5.55, 6.65]
**M3 (Audio + visual)**	**20**	**7.60**	**1.23**	**8.0**	**[7.06, 8.14]**

Note: CI = confidence interval; SD = standard deviation. M1 represents audio only modality. M2 represents visual only modality. M3 represents the combined audio + visual modality.

**Table 4:** Pairwise comparisons between the three detection conditions, with multiple-comparison correction and effect sizes.

Comparison	Mean Diff.	95% CI	*t*	df	*p* Raw	*p* Holm	Wilcoxon *p*	Cohen’s *d*	Magnitude
**Combined vs. M1**	+2.10	[1.51, 2.69]	7.50	19	<0.001	<0.001	<0.001	**1.68**	Very large
**Combined vs. M2**	+1.50	[0.92, 2.08]	5.43	19	<0.001	<0.001	<0.001	**1.21**	Very large
M2 vs. M1	+0.60	[−0.24, 1.44]	1.50	19	0.150	0.150	0.234	0.34	Small (n.s.)

Note: Cohen’s *d* conventions: 0.2 = small, 0.5 = medium, 0.8 = large, and ≥1.2 = very large. Holm refers to the Holm–Bonferroni adjustment for multiple comparisons. n.s. = not significant.

**Table 5:** Repeated-measures ANOVA across the three detection conditions.

Source of Variation	SS	df	MS	*F*	*p*	Critical *F*
Between participants	49.7333	19	2.6175	2.4950	0.0081	1.8673
**Between methods**	**46.8000**	**2**	**23.4000**	**22.3044**	$3.9\times 10^{-7}$	**3.2448**
Unexplained variation	39.8667	38	1.0491	—	—	—
Total	136.4000	59	—	—	—	—

Note: SS = sum of squares; MS = mean square; df = degrees of freedom. Critical *F* is reported for α=0.05.

## Data Availability

Helsinki EEG Dataset: https://zenodo.org/records/2547147 (accessed on 24 June 2026).

## Abbreviations

The following abbreviations are used in this manuscript:

VR	Virtual Reality
2D	Two-Dimensional
3D	Three-Dimensional
CSV	Comma-Separated Values
LOD	Level of Detail
ECG	Electrocardiogram
